# Management of oral Graft versus Host Disease with topical agents: A systematic review

**DOI:** 10.4317/medoral.20968

**Published:** 2015-11-30

**Authors:** Rui Albuquerque, Zahid Khan, Ana Poveda, Jonathan Higham, Andrea Richards, Luis Monteiro, Enric Jané-Salas, José Lopez-Lopez, Saman Warnakulasuriya

**Affiliations:** 1 Oral Medicine Department, Birmingham Dental Hospital/School of Dentistry, University of Birmingham, Birmingham B4 6NN, United Kingdom; 2 Medicine and Oral Surgery Department, Institute of Research and Advanced Training in Health Sciences and Technologies (IINFACTS), Higher Institute of Health Sciences (ISCS-N), CESPU, 4585-116 Paredes, Portugal; 3 Oral Medicine Department. Dental School, University of Barcelona. Pavelló de Govern, 2a planta, Av. Feixa Llarga, s/n, 08907 L’Hospitalet de Llobregat, Barcelona, Spain. Oral Health and Masticatory System Group (Bellvitge Biomedical Research Institute) IDIBELL, L’Hospitalet de Llobregat, Barcelona 08907, Spain; 4 Department of Oral Medicine, King’s College London Dental Institute, WHO Collaborating Centre for Oral cancer/Precancer, Bessemer Road, London SE5 9RS, UK

## Abstract

**Background:**

Oral Graft-versus-Host Disease (oGvHD) is a common complication of haematopoietic stem cell transplantation. Choosing the right topical application to be used intra orally can be a challenge. Consequently, the aim of this work is to review the effectiveness and safety of topical agents currently used in the management of the inflammatory mucosal lesions encountered in oGVHD.

**Material and Methods:**

We carried out electronic searches of publications up to May 2015 of the databases Pubmed, National Library of Medicine’s Medline, Embase and the Cochrane Central Register of Controlled Clinical trials to identify potentially relevant studies (keywords: “oral”, “graft”, “versus”, “host”, “disease” and “treatment”). The main inclusion criterion was the reported use of a topical agent which was not intentionally swallowed when used for the treatment of oGVHD. A 3-point grading system, described by the Swedish Council on Technology Assessment in Health Care and the Centre for Reviews and Dissemination, University of York, was used to rate the methodological quality of the papers.

**Results:**

From the 902 entries identified in the search, 7 studies qualifying for inclusion were analysed. Overall, there is limited evidence with regards to the effectiveness of topical steroids for oGVHD. However, the studies showed some effect of Budesonide alone and when combined with dexamethasone. Topical tacrolimus also appears to have some effect and clobetasol propionate mouthwash had a significantly better clinical response than dexamethasone mouthwash in treating oGVHD.

**Conclusions:**

As the number of clinical trials conducted is limited, there is little evidence to support the use of topical therapies to treat the inflammatory mucosal lesions found in oGVHD. High quality randomised control trials are needed in order to measure the effectiveness of any topical application for the treatment of the inflammatory mucosal lesions found in oGVHD.

**Key words:**Oral, graft versus host disease, topical, therapy.

## Introduction

Graft versus host disease (GVHD) is caused by alloreactive donor T cells, which recognise and target host tissues in immunocompromised recipients (1). It is a common post transplant complication which affects up to half of all haematopoietic stem cell transplant (HSCT) patients (2). The 2005 National Institute of Health (NIH) Consensus Conference first proposed new criteria for the diagnosis of GVHD and scoring the severity of chronic GVHD (cGVHD). In 2014, revisions were made to address areas of controversy or confusion, such as the overlap GVHD subcategory and the distinction between active disease and past tissue damage (3).

Patients with cGVHD may suffer from severe morbidity, usually involving the skin, mouth, eyes, GI tract and liver but other systems such as the lungs, joints and genitourinary tract may also be involved (4). The oral cavity is the second most commonly involved organ system, after skin involvement (4). Oral GVHD (oGVHD) can present as generalised mucosal erythema, erosions, ulcerations, white striae or papules resembling oral lichen planus, and the transplanted patient can also develop oral mucocoeles (4-6). Patients may also complain of xerostomia and pain (5). The use of pre-HSCT chemoradiotherapy conditioning, post-HSCT medications or infections can result in changes in the oral mucosa such as oral mucositis and/or result in oGVHD causing difficulties for clinicians in differentiating between symptoms related to cGVHD and other side effects of therapy (5).

Oral GVHD can be managed with systemic therapy, or topical treatment alone or in combination in order to achieve pain control and provide local palliation (2). Systemic immunosuppression can usually control the oral lesions of cGVHD but patients may require the use of opiates for the control of their pain symptoms (2). Clinically, oGVHD develops during reduction of systemically administered immunusupressants after HSCT. The use of topical agents to control inflammation in oGVHD might allow lower doses or faster reduction in the dose of systemically administered immunusupressants thereby helping to reduce the side effects of systemic therapy (2). We believe a review of the currently reported clinical studies of topical therapies used for the management of the inflammatory mucosal lesions found in oGVHD is advantageous for clinicians in secondary care, to establish the effectiveness of these therapies in the management of this condition. Therefore, the aim of this work is to review the clinical effectiveness of topical interventions for oGVHD and to prepare a clinical guideline for the management of oGVHD or to identify possible gaps in the evidence in order to carry out future research.

## Material and Methods

A systematic review to unify and summarise the published literature on topical agents used for the treatment of the inflammatory mucosal lesions found in oGVHD was undertaken. An electronic search of the databases Pubmed, National Library of Medicine’s Medline, Embase, Cochrane Central Register of controlled clinical trials and Scopus up to May 2015 was undertaken, to identify potentially relevant studies. The key words used in the search included “oral”, “graft”, “versus”, “host”, “disease” and “treatment”.

The inclusion criteria also included randomised controlled clinical trials, cohort studies (retrospective and prospective), and case-controlled studies published in English, Portuguese and Spanish languages for the treatment of oGVHD presenting as inflammatory mucosal conditions such as lichen planus and lichenoid disorders, that included clear descriptions of the topical therapy used and its method of application. The search was limited to human studies. The final criterion was the use of a topical application which was not intentionally swallowed and was used for the treatment of oGVHD. All articles which did not fit into the criteria were excluded, including reviews, author debates, letters to the editor or studies in which it was not clear whether the treatment was for oGVHD or for mucositis secondary to chemotherapy/radiotherapy.

The full papers and abstracts identified through the search engines were independently reviewed by the authors (RA, LM, ZK, AP, JH, SW, AR, EJS & JLL) for inclusion in this systematic review. If there was insufficient information provided in the abstract or if there was a disagreement between the reviewers, the authors reviewed the full text before reaching an agreement through discussion.

**Figure 1:**
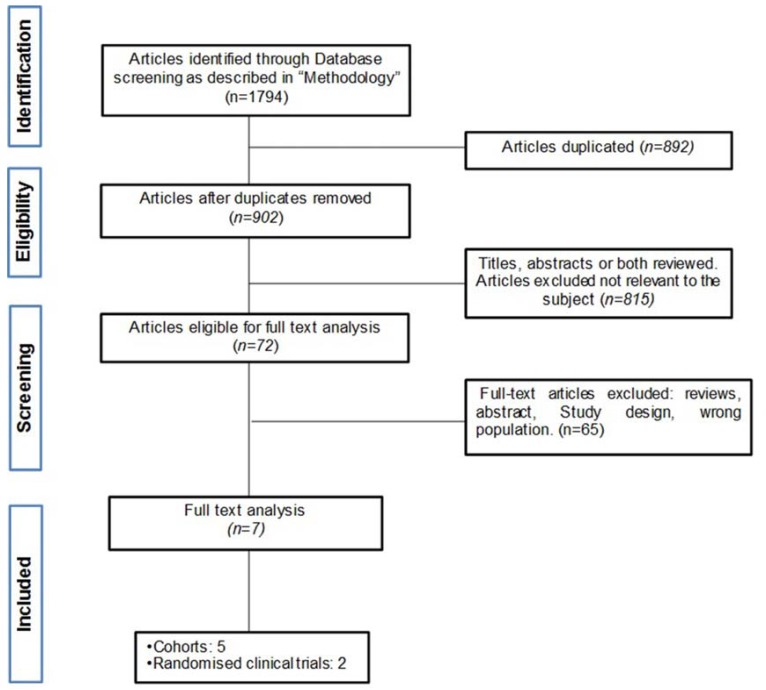
Flow diagram of article selection.

Data extraction was then completed in duplicate by the same independent reviewers. The following data were collected: study characteristics (year, design, number enrolled/analysed, patient median/mean age, intervention, length of follow-up and outcome). The search strategy and the flow diagram of article selection are shown in figure 1.

From those studies, the methodological quality was reviewed as an indication of the strength of evidence provided by the study. Flaws in the design or in the conduction of the study were analysed using PRISMA (Preferred Reporting Items for Systematic Reviews and Meta-Analyses) as well as a 3-point grading system, described by the Swedish Council on Technology Assessment in Health Care (SBU) and the Centre for Reviews and Dissemination, University of York (accessed 30th June, 2015 https://www.york.ac.uk/media/crd/Systematic_Reviews.pdf). With regards to the grading of the studies, this was assessed by all of the authors and any inter examiner conflicts were resolved by discussion of each study to reach a consensus ( Table 1). Other methods to appraise literature have been described with well known advantages and disadvantages (7). This current method has been successfully used in other systematic reviews providing a good opportunity to assess clinical trials and observational studies as reported in other studies (8,9).

**Table 1:**
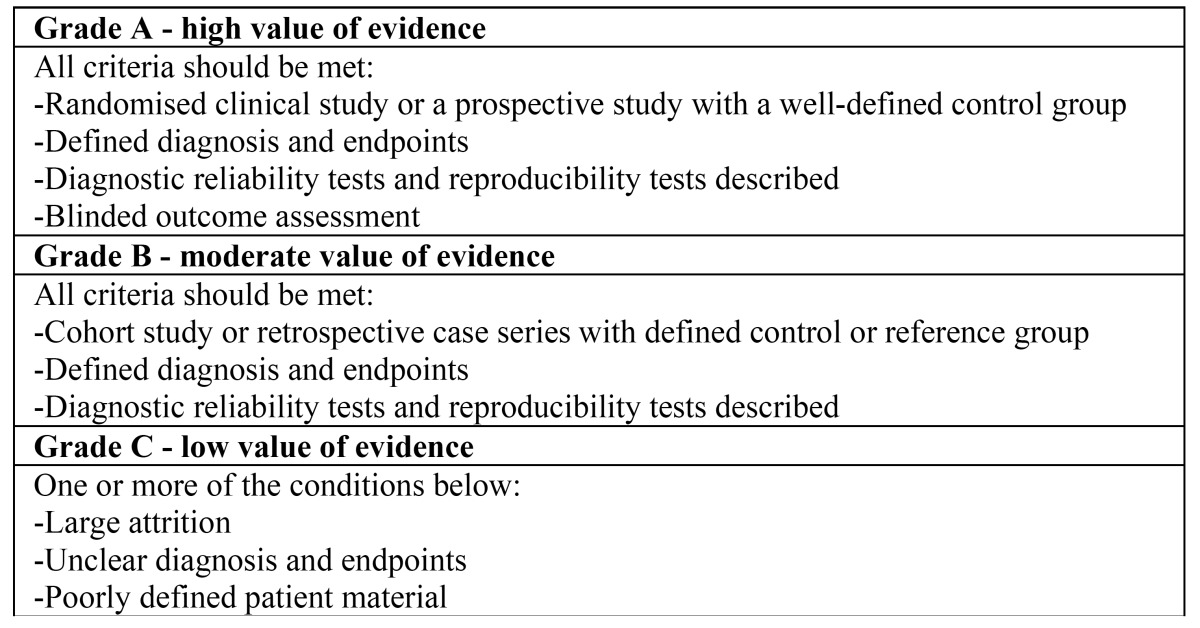
Swedish Council on Technology Assessment in Health Care.

The SBU tool permitted the assessment of the level of the available evidence for reports according to the following grading: Strong: at least two studies of level ‘A’; Moderate: one study of level ‘A’ and at least two studies of level ‘B’; Limited: at least two studies of level ‘B’;

Scarce: fewer than two studies of level ‘B’. Authors (ZK, EJS, JLL) prepared data extraction tables and all authors contributed to summary reports of the selected journal articles, critical appraisal and review of the literature.

## Results

From the 7 studies listed in  Table 2, it is apparent that budesonide, dexamethasone and tacrolimus were the most common topical medications used in published studies. Of the reviewed studies, there were 2 randomised clinical trials (10,11) and 5 cohort studies (12-16). The median age, length of study, topical application, grading and outcome for each study are reported in  Table 2. The side effects reported in each study are listed in  Table 3.

**Table 2:**
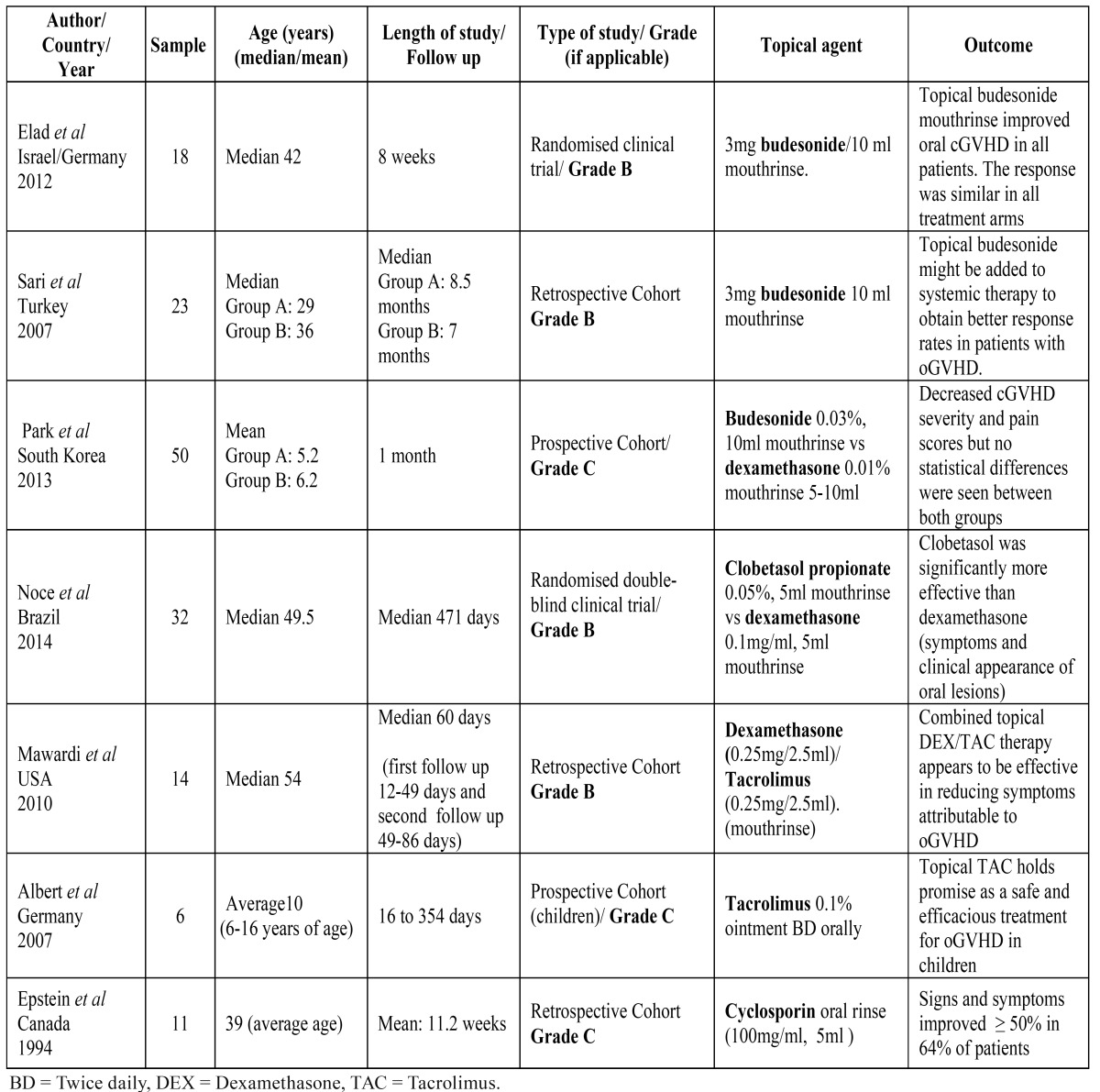
Study reports on the use of topical applications in management of oGVHD.

**Table 3:**
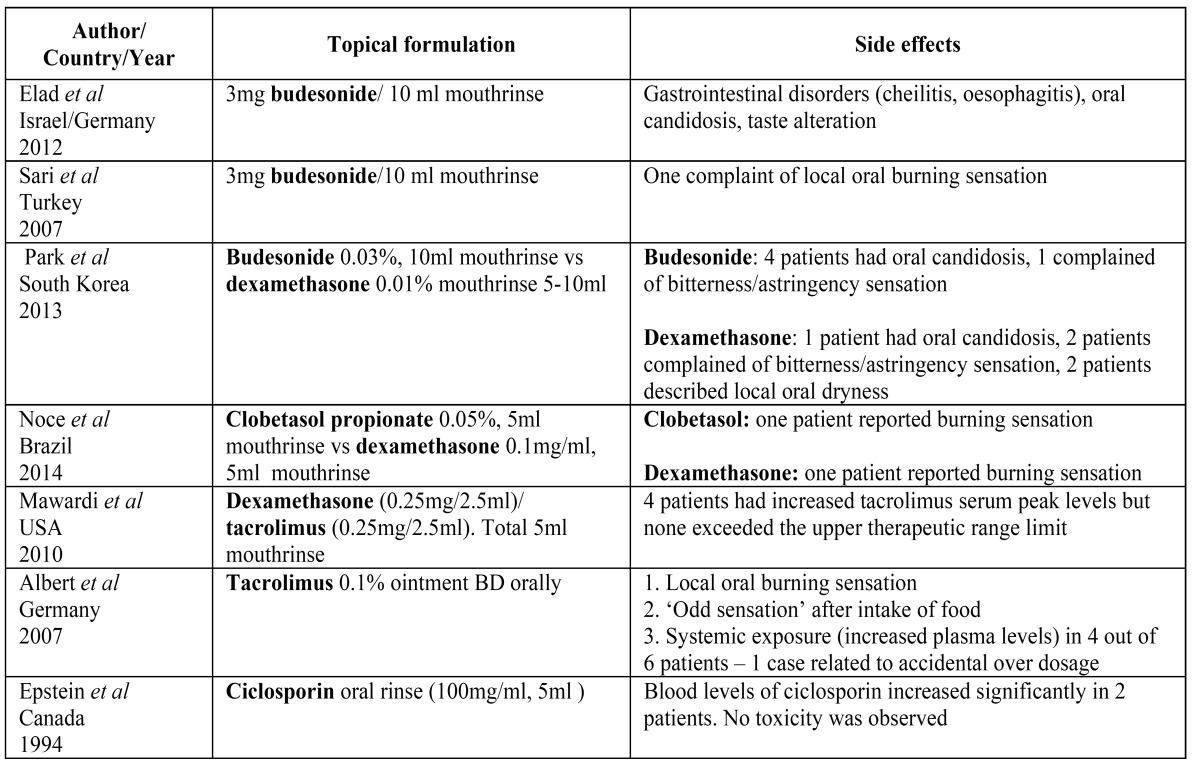
Reported side effects.

The excluded articles were, one case series of the topical use of azathioprine (17) and seven case reports of the use of topical tacrolimus (18-24) ( Table 4). These were excluded as they did not fulfil the SBU criteria.

**Table 4:**
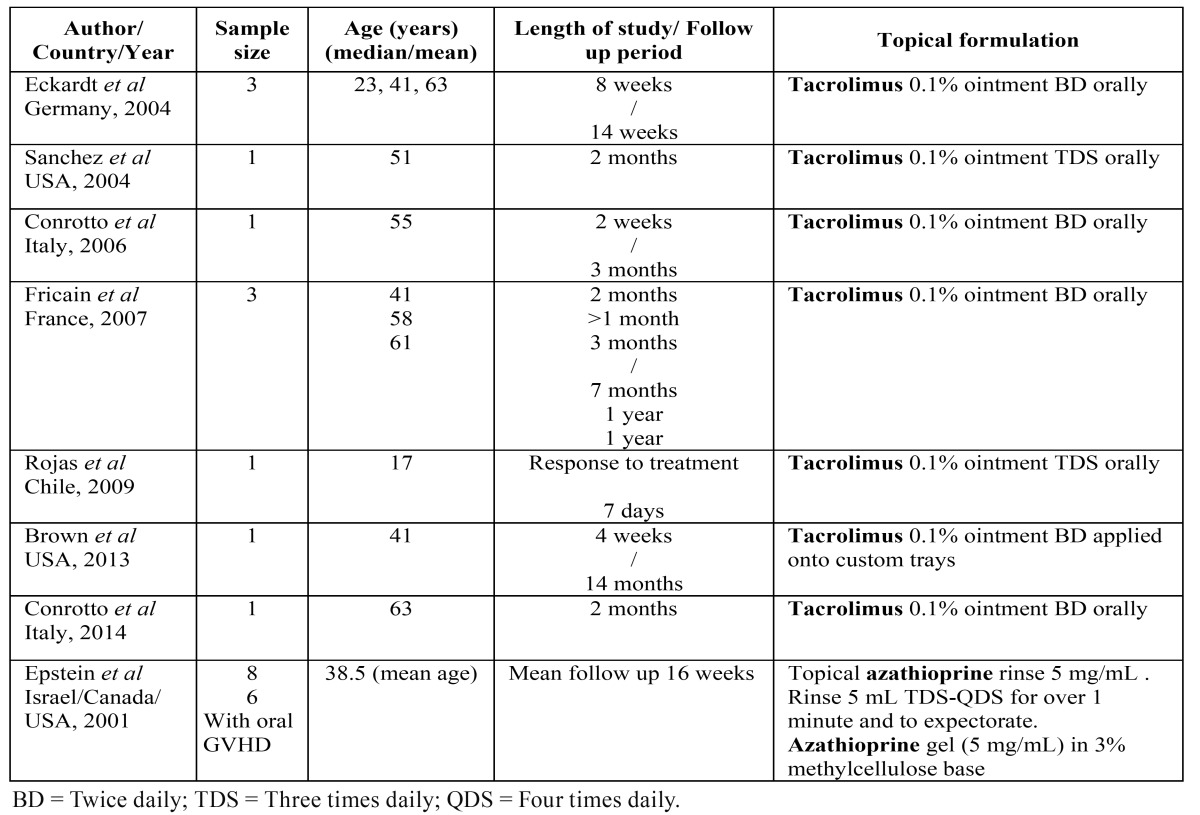
Table of case reports and case series studies: topical applications in patients with oGVHD.

With regards to the scales used for disease assessment in the selected studies, the majority were based on the National Institutes of Health consensus for GVHD to assess the clinical stage of oral manifestations as well as response to treatment (10-12,14,16). Activity of the disease was classified based on the oral mucosa rating scale (OMRS) (10,11) and clinical response graded under complete response, partial response, stable disease or progression (15,16). In some cases, the disease was categorised into 3 stages; mild, moderate or severe (13) and response to treatment labelled as “objective response as an improvement of at least 50% at the final/ withdrawal visit (compared to baseline) in the OMRS” (10).

Subjective evaluation of symptom severity as well as the use of visual analogue scale (VAS) for patient’s self-reporting for oral pain was also used (11,12,16). Albert *et al*. (14) assessed response at baseline and at each visit by asking for subjective evaluation of symptom severity (pain, burning, and discomfort) and by inspection of the oral cavity, assessing the response as complete response, very good partial response, partial response, and non-response. Epstein and Reece (12) evaluated the oral cavity based on the presence of erythema, lichenoid patterns and ulceration, including calculation of the size of any ulcers and the total area of ulceration.

Multiple sources of bias were found in the clinical trials (10,11) such as the lack of adequate description of randomisation. There was a lack of information on who was responsible for generating the randomisation, allocation concealment, assigning participants to interventions, and whether the clinician responsible for the clinical assessment was blinded (10,11). Both studies reported outcomes listed in the methods section and both applied power of the study (10,11). However, both reported limitations of a small sample size, and possible confounding factors such as concomitant immunosuppressive therapies and the duration of topical therapy. In the cohort studies there was a lack of blinding of providers, participants and assessors. Furthermore, intention to treat analysis and power were not used (12-16).

Although Albert *et al*. (14) reported good tolerability and safety as well as potent activity of topical tacrolimus ointment in 6 paediatric patients, their study had many limitations due to the small size and its non-comparative nature. Their response criteria lacked reliability as there was no explanation of who interpreted the results or whether there was more than one rater leading to possible observational bias. In addition, there was no standardised response criteria used at the time of the study, no statistical analysis of results, and possible confounding factors were not identified nor addressed.

Mawardi *et al*. (13) recognised a possible selection bias in their inclusion criteria, such as the selection of severe/refractory cases only of oral cGVHD. This study aimed to describe the clinical outcomes of combined dexamethasone and tacrolimus topical rinses in the management of patients with symptomatic oral cGVHD. Potential confounders and effect modifiers were not clearly defined, and there was a lack of standardisation in the application of the oral solution with its frequency ranging between twice and four times daily amongst patients included in the study.

Sari *et al*. (16) designed a retrospective study according to NIH consensus criteria comparing the use of topical budesonide plus systemic therapy (prednisolone and ciclosporin) compared to those receiving systemic therapy alone. Their results suggested that there may be a potential benefit in the use of topical budesonide as an adjuvant to systemic therapy. This study lacked random allocation and potential confounding factors were not taken into account, hence there were no adjustments to the statistical analysis. The authors commented on this limitation and suggested further studies where randomisation and larger groups should be considered.

Epstein & Reece (12) trial was based on the use of oral ciclosporin rinses in patients with cGVHD who were not responding to systemic therapy and dexamethasone oral rinses, concluding that this may be a possible coadjuvant in refractory cases. However, without the use of standardised response criteria at the time of the study, it is difficult not to interpret the results with caution. The lack of blinding, power sample size, description of the statistical analysis of the results, and no effort to address potential sources of bias limit the impact and application of this study.

Park *et al*. (15) compared the efficacy of topical budesonide relative to dexamethasone treatment, with there being no statistical differences found between the two groups. This was a well-structured study where statistical analysis of the results were reported and interpreted appropriately. Unfortunately, this study had no power calculation and possible confounders were not adjusted for. Finally there was the limitation of a short follow up period in this study.

We present a resume of the effectiveness of the topical interventions used in the selected studies:

Randomised clinical trials:

There were two clinical trials included in this review, neither study included a control group, one study evaluated the effectiveness of budesonide mouthwash (10) and the other compared the effectiveness of clobetasol propionate mouthwash vs dexamethasone mouthwash (11). A third trial will also be mentioned despite being presented as a letter to the editor evaluating the use of topical thalidomide gel (25).

Elad *et al*. 2012 (10) in a double blind clinical trial, divided 18 participants into four groups that used budesonide 3mg as a mouthwash twice daily for 5 or 10 minutes compared to two other groups who used the mouthwash three times daily for either 5 or 10 minutes. The participants enrolled all completed the 8 week study period. The oral lesions improved in all patients, but the rate of objective improvement (defined as ≥50%) and pain reduction was not significantly different between the 4 study arms (*P* <0.05).

Noce *et al*. 2014 (11) in an open randomised trial, enrolled 32 participants who were divided between study groups that received either clobetasol propionate (0.05%) mouthrinse or dexamethasone (0.1mg/5ml) mouthrinse. The median length of the study was 471 days. The use of clobetasol propionate (0.05%) resulted in a significant reduction in the oral mucosa rating scale total score (*P* = 0.04) and in the ulcer score (*P* = 0.03) when compared with topical dexamethasone. In both groups, there was significant symptomatic improvement but the response was significantly greater in the clobetasol group (*P* =0 .02).

John *et al*. 2013 (25) reported a phase II, randomised, placebo-controlled, double-blind clinical trial as a letter to the editor. When including this study for analysis using the 3 point grading system, the amount of information was restricted and therefore did not allow for fair grading. They recruited 10 participants and compared the use of topical thalidomide 20mg gel against a placebo controlled group in patients with oGVHD. The length of the study was 4 weeks, with an 8 week follow up period. Only 6 subjects completed the study, 3 in each group. The authors recognised the small sample size precluded statistical confirmation of topical thalidomide’s efficacy in the management of oral ulcerative cGVHD, They also considered that the placebo effect of the Orabase plain product and/or the waxing and waning nature of cGVHD might impact on their findings. Overall, there was a 66% mean percentage decrease in the total surface area of oral ulceration at week 4 compared with the baseline presentation. It was also observed that the TNFα levels in saliva and ulcer exudates decreased with therapy but with no significant difference in the salivary levels of TNFα compared with baseline in both thalidomide (*P*=1.0) and placebo (*P*=0.109) groups. However, there was a decrease in salivary IL6 concentration in the thalidomide group (*P*=0.465) when compared with the baseline, with no decrease in the placebo group.

- Cohort studies:

Park *et al*. 2013 (15) enrolled 50 participants, dividing them into two groups and compared the use of budesonide 0.03% oral rinse with 0.01% dexamethasone rinse. All participants completed the one month study period. No statistical difference in the response was noted between the two groups (*p* = 0.093). However, recently, in 2014, Zadik*et al*. (26), carried out a reanalysis of the reported data, excluding patients with a (NIH) consensus criteria score of 1 and 2, considered not to be fully eligible for topical steroids, and a further statistical analysis revealed a significantly better response to budesonide than to dexamethasone (*p* = 0.015).

Albert *et al*. 2007 (14) recruited six children (aged 6-16 years) and presented a cohort study reporting the responses of children treated with topical tacrolimus 0.1% ointment. The first response to treatment was documented after a median of 21 (range 6-31) days. The follow up period ranged from 16 to 354 days. A complete response was achieved in two of the six patients with a partial response in the remaining four patients.

Mawardi *et al*. 2010 (13) enrolled 14 subjects looking at the efficacy of combined topical dexamethasone (DEX) and tacrolimus (TAC) solutions in the management of oral cGVHD.

The median length and follow up of the study was 60 days. The study showed a median improvement in the NIH total score at the second follow up visit (range from 2 to 6, *P*=0.06), though unchanged at the first follow up (range from 2 to 5, *P*=0.41). Both erythema and lichenoid feature scores demonstrated a statistically significant improvement at the second follow up. Mawardi *et al*. (13) recognised the selected cases could be biased by the type of disease that they could include in the study.

Epstein and Reece 1994 (12) recruited 11 participants and evaluated the use of ciclosporin A (100mg/ml) administered as an oral rinse (5ml) in patients with active oral lesions, despite the concurrent use of systemic immunosuppressants plus topical dexamethasone. The mean length of the study was 11.2 weeks. The study showed an improvement in ulceration of ≥ 50% in 7 of 11 patients (64%) treated with the addition of topical ciclosporin A.

Sari *et al*. 2007 (16) divided 23 participants into two groups of which one group received topical budesonide (3mg in 10 ml) oral rinse in addition to systemic therapy (prednisone and ciclosporin) (Group A, n = 12), compared to another group of subjects who were administered the systemic therapy alone (Group B, n = 11). The median length of the study was 8.5 months and 7 months for groups A and B respectively. The median follow up period was 108 days for group A and 120 days for group B. There was an overall clinical response rate of 83% and 36% for groups A and B, respectively (*P* = 0.036). However, no statistically significant differences were found between median pain scores of the two groups before and after treatment (*P* = 0.740 and *P* = 0.091).

## Discussion

Based on this systematic review, the number of randomised clinical trials reporting the use of topical steroids or immunosuppresants in the management of the inflammatory mucosal lesions encountered in oGVHD appears to be very low. Using the Swedish Council on Technology Assessment in Health Care and the Centre for Reviews and Dissemination, University of York 3- point grading system, there is limited evidence to support these therapies based on the analysed papers. The reported studies presented different lengths of follow up and also differently scaled schemes of assessment of outcome. The sample sizes were generally small, however both clinical trials provided a statistical power of 80%, a commonly accepted value (10,11). In all of the selected studies it is not recorded whether the outcome assessors were blinded when assessing the response to treatment. This bias could have been prevented by ensuring outcome assessors are blind to treatment allocation, for example by using independent clinicians who are not otherwise involved in the trial to assess patients, or using a blinded adjudication committee to determine the outcome (27). However, the nature of the study can preclude this, as reported by Kahan *et al*. (28) who reported that a “blinded outcome assessment may not always be feasible due to the nature of the trial, for example, when all researchers in a centre are aware of treatment allocation, or relevant clinical information cannot be sent to a blinded assessor”.

Currently, the literature search in systematic reviews and the quality assessment of included studies are well-established processes in evidence-based medicine and dentistry (29). However, the precise methods for the process can differ among various systematic reviews (7). Meier *et al*. (6) (2011), provided a review of topical applications used for GVHD. We believe this new systematic review could provide new data, acknowledging observational studies as well as clinical trials. We included five cohort studies in addition to two clinical trials based on the SBU guideline. We acknowledge that one of the limitations of this review is that the search methodology/parameters used in this review cannot guarantee that all articles pertinent to the topic were included; other databases may include information in this field. Apart from English, we included Portuguese and Spanish language studies, however we acknowledge that searches including other languages may have returned relevant articles and further reviews including other languages might be considered.

A notable finding was the lack of randomised controlled trials in the effectiveness of topical therapy in the management of oGVHD. The randomised controlled trial is acknowledged to be the most powerful tool to evaluate the effectiveness of treatment and the quality of the study significantly affects the validity of the conclusions drawn (9). John *et al*. (25) showed thalidomide (20mg gel) was effective in individuals with oral cGVHD, although the authors did recognise the potential influence of the small size of the sample in the reporting of their results. As this was presented as a ‘letter to the editor’, there was a lack of detailed description of randomisation, allocation, concealment and blinding, and as such the conclusions drawn from these data need to be interpreted with caution. However, we felt that the nature of this study was relevant to this review and as such its inclusion was warranted. The lack of randomised control trials on this topic could be due to practical difficulties secondary to the nature of patients with oGVHD, which can raise both ethical and logistical issues.

The majority of the studies had a cohort design including two retrospective cohorts and two prospective cohorts. From an evidence-based point of view, the scientific value of a retrospective study is limited although some authors have argued that well-designed prospective or retrospective studies should not be ignored when assessing the available scientific literature (8).

The majority of patients in the selected studies were also taking systemic medications along with the topical application studied. The intention was to analyse the effect of the topical applications on the disease. As a result it is difficult to ascribe any improvements described to the systemic medication or the topical therapies. As such these results should be interpreted with caution. Dilger. *et al*. (30) report that in patients with oral cGVHD, 10% of the topical dose of budesonide can enter the circulation, possibly due to the loss of epithelial integrity that can occur in ulcerative lesions in oGVHD and metabolisation. When compared with the systemic concentrations attained after the oral intake of enteric-coated budesonide for inflammatory bowel diseases, the levels reported are within recognised safety thresholds.

A multicentre questionnaire undertaken by Elad *et al*. (31) regarding the treatment of oral cGVHD showed that steroids were the first-line topical management of choice (91.7 %). The topical steroids budesonide (3mg/10ml) and dexamethasone (0.1mg/5ml) were the most commonly studied. Tacrolimus (TAC) was studied as a single topical application or in combination with dexamethasone. TAC bonds to the immunophilin FK506 binding protein, inhibiting calcineurin phosphatase. It inhibits calcium-dependent events, such as interleukin-2 gene transcription, nitric oxide synthase activation, cell degranulation, and apoptosis (32).

With regards to the side effects and safety, a cohort study undertaken by Mawardi *et al*. (13) found that when topical DEX and TAC were used in combination, 4 patients (37%) had increased TAC serum peak levels that corresponded with the application of topical treatment. Following the use of topical TAC, many reports have raised concerns on the clinically significant elevation of tacrolimus serum levels (13,22). Some case reports have shown elevated levels of TAC although the actual absorption via the oral mucosa remains ambiguous (14,22). A retrospective study by Thomson *et al*. (33) described the safety of TAC when used to treat individuals with erosive lichen planus. They noted minor local side effects but an absence of evidence of systemic absorption.

As has been frequently reported there is an increased risk of oral malignancy in oral GVHD (5), although to our knowledge, this increased risk of malignancy has not been associated with the use of topical tacrolimus in cases of oral GVHD. Another calcineurin inhibitor; ciclosporin, has been studied for the treatment of continuous oral ulceration used as both a topical and systemic agent. Epstein & Reece (12) showed improvements in oGVHD using topical ciclosporin. Many studies on mucosal inflammatory conditions such as lichen planus did not report improved outcomes with the use of this calcineurin inhibitor when compared with topical steroids (34). The appropriate follow up time to assess the response to treatment is debatable, particularly with regards to the monitoring of potential oral malignancy (35). The increased risk of secondary malignancies is a major late complication of HSCT. A study showed that 2-6% of post-HSCT patients had developed a secondary solid tumour within 10 years (35). Approximately one-third of these secondary solid tumours were squamous cell carcinomas (SCCs) of the skin or the oral cavity. The oral cavity accounted for up to half of the SCC cases alone. Development of malignancy was not reported during the short length of the included studies and longer follow up periods could be considered with this in mind. Curtis *et al*. (36) reported that in 16 cases of oral SCC associated with oGVHD, the most frequent site of SCC was the tongue, followed by the lips, and gingivae. From the 16 cases, 11 had received immunosuppressive drugs for two or more years and the risk of SCC in the oral cavity appears to be higher in patients who have undergone HSCT.

The use of budesonide has also been studied by Elad *et al*. in an open label trial (37), involving twelve patients diagnosed with chronic and resistant oral GVHD. They were managed with topical budesonide (3 mg /5 ml saline) 2 to 3 times a day for up to 3 months. Their findings showed that 7 of the 12 patients had scores of “good” or “complete” recovery following evaluation by the examiner and assessment of the subject. In some patients in this study, the systemic management with both prednisolone and ciclosporin was gradually reduced as early as 2 weeks following the commencement of budesonide mouthwashes. The authors stated that 3 examiners were used to carry out the clinical examinations and recognised that inter-observer calibration was not implemented. Van Schandevyl *et al*. (38) have presented a protocol where budesonide mouthwash was used as an adjuvant (Budenofalk® 3 mg gastro-resistant capsules) and results of a large-scale, phase III, randomised, controlled, double-blinded multicenter study assessing this new preparation of topical budesonide for the treatment of oral cGVHD is awaited.

Noce *et al*. (11) compared the use of topical clobetasol with dexamethasone, demonstrating significant symptomatic improvements over baseline in both groups, but with the response being significantly greater in the clobetasol group. The authors recognised the limitations of this study such as the small number of patients enrolled and other confounding variables, and suggested that further studies with larger samples and subject stratification, taking into account the influence of these variables on the topical therapy for oral cGVHD lesions, might be considered. Erosive oral lichen planus (OLP) has close similarities in its disease presentation to oGVHD. It is therefore pertinent to consider what topical treatments are used for the management of erosive OLP. In a Cochrane review of topical applications for the treatment of oral lichen planus, the authors concluded there is no evidence that any one topical steroid is more effective than another (39). Different treatment regimens using clobetasol have been proposed in the literature (40). Previous studies have used 0.05% topical clobetasol applications, 2 or 3 times a day, for a period between 2 weeks and 2 months (11,39,40). Topical dexamethasone has been used in a 1 mg/mL solution or in a 0.043% paste, 3 to 4 times a day, for 1 month (11). Reported symptomatic improvement of erosive oral lichen planus lesions varied from 63.6% to 100% with topical clobetasol, and from 38.50% to 72.51% with topical dexamethasone (11,39,40).

Budesonide was potentially linked to some side effects including gastrointestinal tract disorders (cheilitis, oesophagitis), oral candidosis and taste disturbance (10). Similar findings of side effects were reported in other studies using budesonide, as well as oral burning sensations (15,16,37). In studies where dexamethasone mouth rinse was used, side effects were reported including oral burning sensations, oral candidosis, local dryness and bitterness/astringency sensations (11,15). A local oral burning sensation was reported by one patient using topical clobetasol propionate (11) ( Table 2).

In conclusion, the evidence for the use of topical therapies in the management of the inflammatory mucosal lesions encountered in oGVHD is limited and the results of this review should be interpreted with some caution. Oral GVHD is a very debilitating condition particularly with respect to the effect on quality of life (QOL). We suggest that high quality randomised controlled trials of the effectiveness of topical therapies in oGVHD, with good statistical power and standardised outcome measures are needed to establish the effectiveness of both established and novel therapies in order to allow the development of clinical guidelines for the topical management of the inflammatory mucosal lesions found in oGVHD.
